# Impact of left ventricular late enhancement on pulmonary arterial hypertension in idiopathic dilated cardiomyopathy

**DOI:** 10.1186/1532-429X-17-S1-P286

**Published:** 2015-02-03

**Authors:** Esther Pérez-David, Manuel Martinez-Sellés, Raquel Yotti, Javier Bermejo, Maria Luisa Sánchez Alegre, Jesús Jiménez Borreguero, Maria José Olivera, Gerard Loughlin, Francisco Fernandez Avilés

**Affiliations:** Cardiology, Hospital Gregorio Marañón, Madrid, Spain; Radiology, Hospital Gregorio Marañón, Madrid, Spain; Cardiology, Hospital La Princesa, Madrid, Spain; Radiology, Hospital La Princesa, Madrid, Spain

## Background

Fibrosis determined by late enhancement (LE) is a predictor of progressive heart failure (HF) in dilated cardiomyopathy (DCM). The mechanism responsible for this association is not fully understood, though increased ventricular stiffness could be involved. We therefore hypothesised that pulmonary vascular resistance (PVR) should be increased in patients with DCM and LE.

## Methods

71 consecutive patients (p) with DCM, left ventricular systolic dysfunction (LVEF<35%) and normal coronary angiography followed in an outpatient HF clinic, were prospectively enrolled in two institutions. All p had to be in stable clinical condition in the last month. Exclusion criteria were: contraindications for contrast-enhanced cardiac MR (ce-CMR), significant impairment of lung function by clinical criteria or spirometry and history of thromboembolic disease. All patients underwent ECG, echo, blood test and a ce-CMR study in a Philips Intera ® 1.5 T scanner, which included cine imaging, phase contrast in the main pulmonary artery and aorta and late enhancement (LE). Postprocessing was performed with QMASS 7.2 ® (Medis, The Netherlands). PVR was calculated following the equation: 19.38 -(4.62*Ln pulmonary artery average velocity) - (0.08 x RVEF %)

## Results

Mean age was 61±12 years, 28 p (54%) were male. 41 p (58%) were in functional class II and 10 p (14%) in class III. 68 p(96%) were on beta-blockers, 61(86%) on ACE inhibitors and 15 (21%) on angiotensin II receptor antagonists. Mean LVEF was 29±10%. LE was normal in 37 p (52%), mesocardial in 23 p (39.6%), and subendocardial in 5 p (7%). In a multivariant regression model which included age, sex, LVEF, E/A ratio and the presence of any LE, the last three parameters were independent predictors of PVR.

## Conclusions

The presence of LE on CMR is a predictor of increased RVP, showing complimentary value to LVEF.

## Funding

This study was supported by a grant from the *Fondo de Investigación Sanitaria*, *Instituto Carlos III*, *Madrid*, *Spain*.

**Table 1:** Independent predictors of PVR

	B (CI 95%)	p
Age	0.02 (-0.04 - 0.08)	NS
sex	0.05 (- 1.10 - 1.20)	NS
E/A ratio	1.10 (0.15 - 2.05)	0.03
LVEF (%)	-0.09 (-0.14 - (-0.03))	0.01
LE (any)	2.08 (0.80 - 3.36)	0.003

